# The Impact of Left Atrium Appendage Morphology on Stroke Risk Assessment in Atrial Fibrillation: A Computational Fluid Dynamics Study

**DOI:** 10.3389/fphys.2018.01938

**Published:** 2019-01-22

**Authors:** Alessandro Masci, Lorenzo Barone, Luca Dedè, Marco Fedele, Corrado Tomasi, Alfio Quarteroni, Cristiana Corsi

**Affiliations:** ^1^DEI, University of Bologna, Bologna, Italy; ^2^MOX, Mathematics Department, Politecnico di Milano, Milan, Italy; ^3^Department of Cardiology, Santa Maria delle Croci Hospital, AUSL della Romagna, Ravenna, Italy

**Keywords:** left atrial appendage, computational fluid dynamics, thrombogenicity, atrial fibrillation, stroke risk assessment

## Abstract

Atrial fibrillation (AF) carries out a 5-fold increase in stroke risk, related to embolization of thrombi clotting in left atrium (LA). Left atrial appendage (LAA) is the site with the highest blood stasis which causes thrombus formation. About 90 % of the intracardiac thrombi in patients with cardioembolic events originally develop in the LAA. Recent studies have been focused on the association between LAA anatomical features and stroke risk and provided conflicting results. Haemodynamic and fluid dynamic information on the LA and mostly on the LAA may improve stroke risk stratification. Therefore, the aim of this study was the design and development of a workflow to quantitatively define the influence of the LAA morphology on LA hemodynamics. Five 3D LA anatomical models, obtained from real clinical data, which were clearly different as regard to LAA morphology were used. For each LAA we identified and computed several parameters describing its geometry. Then, one LA chamber model was chosen and a framework was developed to connect the different LAAs belonging to the other four patients to this model. These new anatomical models represented the computational domain for the computational fluid dynamics (CFD) study; simulations of the hemodynamics within the LA and LAA were performed in order to evaluate the interplay of the LAA shape on the blood flow characteristics in AF condition. CFD simulations were carried out for five cardiac cycles. Blood velocity, vorticity, LAA orifice velocity, residence time computed in the five models were compared and correlated with LAA morphologies. Results showed that not only complex morphologies were characterized by low velocities, low vorticity and consequently could carry a higher thrombogenic risk; even qualitatively simple morphologies showed a thrombogenic risk equal, or even higher, than more complex auricles. CFD results supported the hypothesis that LAA geometric characteristics plays a key-role in defining thromboembolic risk. LAA geometric parameters could be considered, coupled with the morphological characteristics, for a comprehensive evaluation of the regional blood stasis. The proposed procedure might address the development of a tool for patient-specific stroke risk assessment and preventive treatment in AF patients, relying on morpho-functional defintion of each LAA type.

## 1. Introduction

Atrial Fibrillation (AF) is the most common type of arrhythmia. It was demonstrated that the lifetime risk of developing AF after 40 years of age is 26% for men and 23% for women of European descent (Briceno et al., 2015; Zakeri et al., 2017). Over 6 million Europeans suffer from AF and its prevalence is estimated to at least at least double in the next 50 years as the population ages. of stroke 5-fold (January et al., 2014). LAA is the remnant of the embryonic left atrium where the smooth-walled LA originates from the primordial pulmonary vein and its branches (Reddy et al., 2014). Because of its hooked morphology, the LAA is the left atrial site of the highest blood stasis risk, increasing the incidence of thrombus formation and stroke. In fact, 90 % of the intracardiac thrombi in patients with cardioembolic stroke/TIA are considered as originating in the LAA (Yaghi et al., 2015). Oral anticoagulation therapy was the only option available until recently. However, it increases bleeding risk and interpheres with other drugs and multiorgan functioning, and its risk can overtake the otherwise remakable benefits on thromboembolic events. (Hankey and Eikelboom, 2011).

For these reasons, different strategies have been developed such as the use of interventional treatments, i.e., LAA percutaneous closure, which seems to better reduce the risk of thromboembolism compared to warfarin anticoagulation therapy (Reddy et al., 2013). These treatments are restricted to small subgroups of patients, due to the procedural risks and costs which may overcome the preventive antiembolic efficacy.

Therefore, morphological and quantitative features of LAA have been increasingly studied in the past few years. Di Biase et al. (2012) presented a classification of LAA morphology into four different classes (chicken wing, cauliflower, windsock, and cactus) based on their morphological complexity. They studied the correlation between this characteristic and the patient-specific stroke history. Results demonstrated that chicken wing LAA morphology, which is the most prevalent, was less likely to have an embolic event compared to the other LAA types.

Jeong et al. (2016) investigated whether the morphometric or volumetric parameters of LAA would be related to the development of cardioembolism in subjects with AF. They found that LAA orifice diameter and LAA volume were larger in patients with a history of cardioembolic stroke with respect to the control group. AF determines alterations in the LAA wall and surrounding structures and this could explain why elevated LAA volumes are found in stroke patients. Despite these attempts, the association between the aforementioned LAA anatomical features and stroke risk does not seem straightforward, and conflicting results have been published. Moreover, it is still uncertain what is the best strategy for stroke prevention in AF. To this purpose, several clinical studies suggested that stroke risk stratification could be improved by using hemodynamic information on the left atrium (LA) and mainly on the left atrial appendage (LAA) (Gupta et al., 2013; Markl et al., 2016). Computational fluid dynamics (CFD) represents a valuable non-invasive tool to determine and assess meaningful biophysical indicators in a complex fluid dynamics systems, such as velocity, pressure fields, cardiac blood flowrates, vorticity, turbulent kinetic energy, etc. To date, few contributions are based on the CFD modeling of the LAA (Koizumi et al., 2015; Otani et al., 2016; Olivares et al., 2017; Bosi et al., 2018). These studies are more focused on the correlation between LAA hemodynamic characteristics and morphology type in order to compare the results obtained with the observations by Di Biase et al. (2012).

To the best of our knowledge, the interplay of the geometric parameters such as LAA length, tortuosity, surface area and volume with the fluid dynamics parameters have not been investigated. This analysis might highlight new insights into the effects of atrial fibrillation on the LAA haemodynamics, thus providing a better understanding of the thrombi formation risk. Moreover, previous studies considered not only different LAA morphologies for the CFD simulation but also different LA shapes which varied for each patient analyzed. Therefore, considerations regarding the LAA fluid dynamics and mainly its thrombogenicity could be affected also by the variation of the other LA anatomical structures.

The aim of this study was the design and development of a workflow to quantify the influence of LAA morphology alone on the LA hemodynamics. Five 3D LA (including LAA) anatomical models, obtained from real clinical data (CT (CT and MRI), which were all different in regards to the LAA each LAA we computed several parameters describing its geometry. Then, one LA chamber model was chosen and a framework was developed to connect the different LAAs belonging to the other four patients to this model. These anatomical models represented the computational domain for the CFD model described in Masci et al. (2017). Simulations of the haemodynamics within the LA and LAA in AF conditions were performed. In this paper we discuss the details of the developed pipeline. A quantitative analysis for each LAA was performed before the CFD simulations by evaluating the most significant geometrical parameters. Afterwards, the association between geometrical and fluid dynamics indexes were computed in order to evaluate, for each LAA, the correlation with the blood stasis risk and thrombi formation.

## 2. Methods

**Figure 1:**
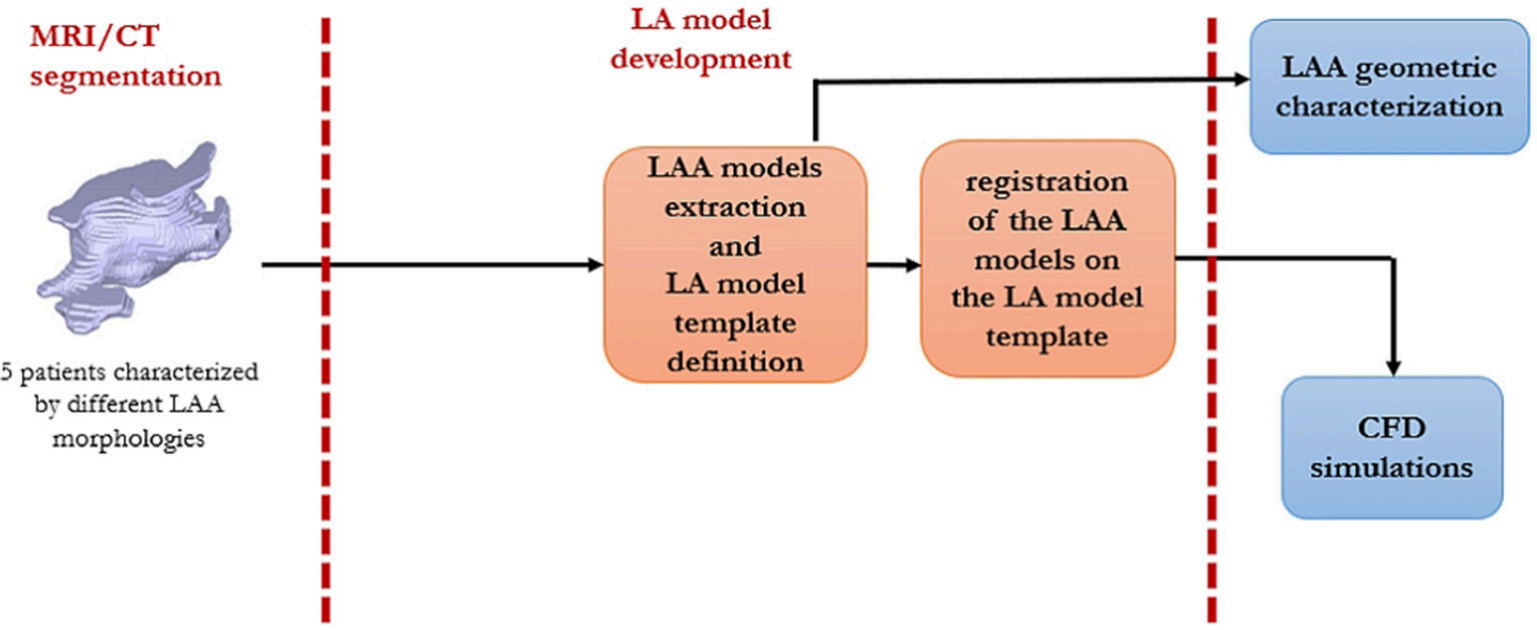
A schematic description of the workflow of this study.

A schematic workflow of the project is shown in Figure 1.

### 2.1. Patients Data

The data-set consisted of five LA 3D anatomical models. These models were extracted from CT (one) and MRI (four) data, in patients affected by AF. The study was approved by the Ethics IRST, IRCCS AVR Committee (CEIIAV n. 1456 prot. 6076/2015 I.5/220). All subjects gave written informed consent in accordance with the Declaration of Helsinki.

Data were processed with specifically designed in-house image segmentation algorithms, described in Valinoti et al. (2018); Masci et al. (2017). From the 3D LA binary masks, we generated the surface meshes by using the MATLAB iso2mesh toolbox (Fang and Boas, 2010).

### 2.2. LAA Extraction

**Figure 2:**
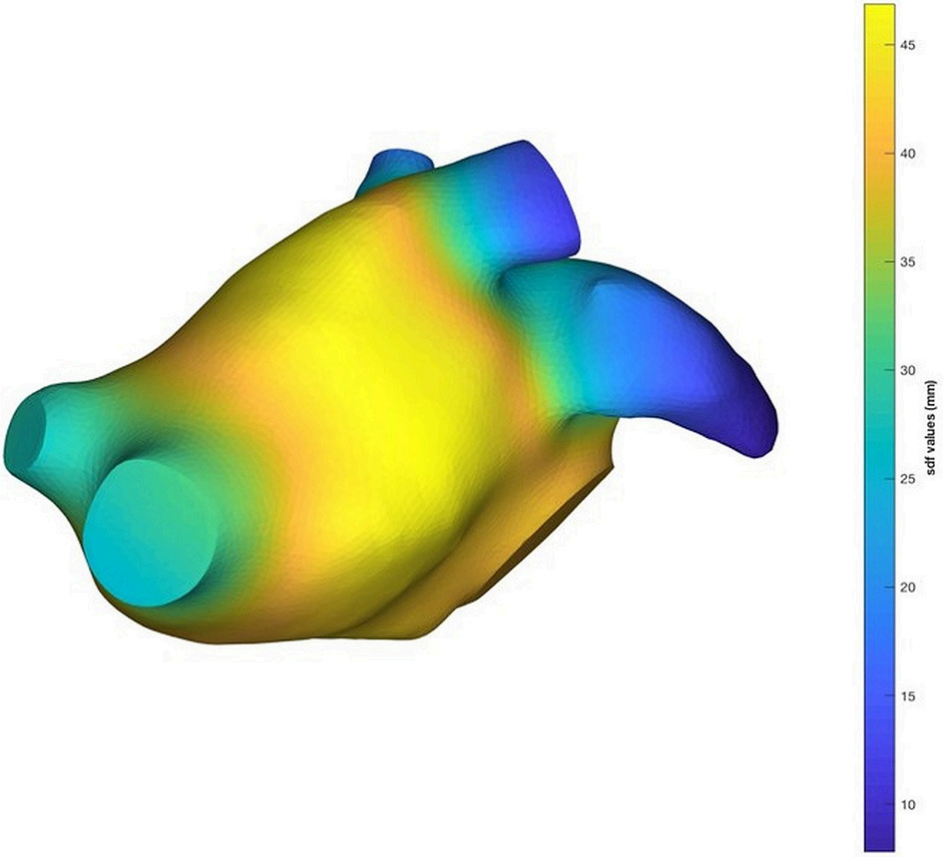
An example of the SDF result on a LA mesh.

The next step was focused on the implementation of an algorithm which automatically recognized and isolated the LAA from the data-set of left atrial anatomical models. To this purpose, the shape diameter function (SDF) proposed by Shapira et al. (2008) was employed. SDF defines a scalar function on the mesh surface *M* (*f*_*s*_ : *M* → ℝ) that measures the local object diameter. From a point *p*, a cone of rays was sent toward the interior of the mesh in the opposite direction to the normal at *p* and the distance between *p* and the intersection of each ray with the mesh was computed. In order to remove false intersections, the intersections with normals at the point of intersection in the same direction as *p*, were removed. Furthermore, only the rays whose length fell within one standard deviation from the median of all lengths were considered. The final SDF value at the point *p* was defined as the weighted average of the remaining lengths, where the weight values were the inverse of the angle between ray and the center of the cone. In this study, the SDF was calculated for each 3D surface using the CGAL software (Fabri and Teillaud, 2011). An example of the SDF values computation for one LA anatomical model is depicted in Figure 2.

**Figure 3:**
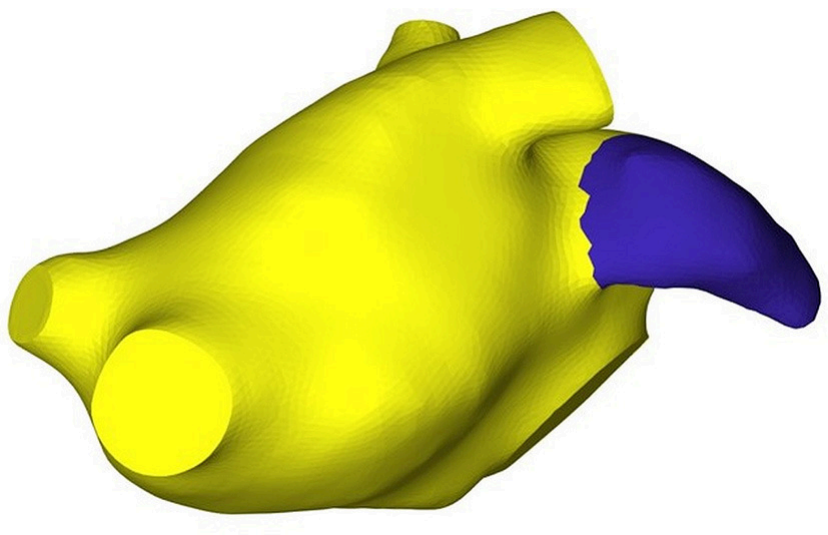
LAA vertices and faces detection.

The SDF values created iso-contours on the mesh which were used to separate regions with different SDF values. Based on this idea, we obtained a semantic clusterization of our 3D models where each cluster had a different id number. The output of this procedure was a vector that contained a label for each facet. This step allowed the identification of pulmonary veins (PVs), LA chamber and LAA for each mesh. In order to identify the LAA, we detected the atrial chamber identificator by calculating the mode (statistics) of the label vector; afterwards we assigned to the PVs the same identificator of the LA chamber. Based on the anatomical position of the LAA we were able to select this region of interest in order to automatically identify it and isolate from the LA surface mesh. An example of the result of this step is shown in Figure 3.

Applying this approach, we obtained five LAA surface meshes. Once the LAA was removed, the LA mesh shown in Figure 2 was used as template for the definition of the new LA anatomical models, each one was characterized by a particular LAA morphology.

### 2.3. LAA Alignment and Definition of the New LA Models

Once the previous steps were performed, in order to accomplish the alignment between the LA template and the LAA meshes, we identified the vertices which belong to LAA ostia and the LA ostium template. The output was a vector that contained the detected vertex coordinates. The local correspondence between LAA and LA template ostium vertices was achieved by using the iterative closest point (ICP) algorithm (Besl and McKay, 1992). The ICP algorithm matched closest points between two point data sets: one was used as a fixed data set and the other as a floating data set. In our case, the fixed one was composed by vertices of the LA ostium and the floating one by the LAA ostium vertices.

The ICP algorithm iteratively performed the following steps:

1. Matching: for each vertex of the floating data set, the nearest neighbor vertex of the fixed data set was found;
2. Minimization: the error metric was minimized between these two set of vertices;
3. Transformation: floating data points were then mapped in the new space using the computed transformation.

Once this procedure was employed, a set of five LA anatomical models, presenting the same atrial chamber but different LAA geometries, was created.

Finally, in order to refine the anatomical models and to comply with the requirement of providing a smooth geometrical representation of the computational domain for the CFD simulation, a Laplacian smoothing filtering and Poisson surface reconstruction were applied by using MeshLab (Cignoni et al., 2008).

### 2.4. LAA Geometrical Parameters

**Figure 4:**
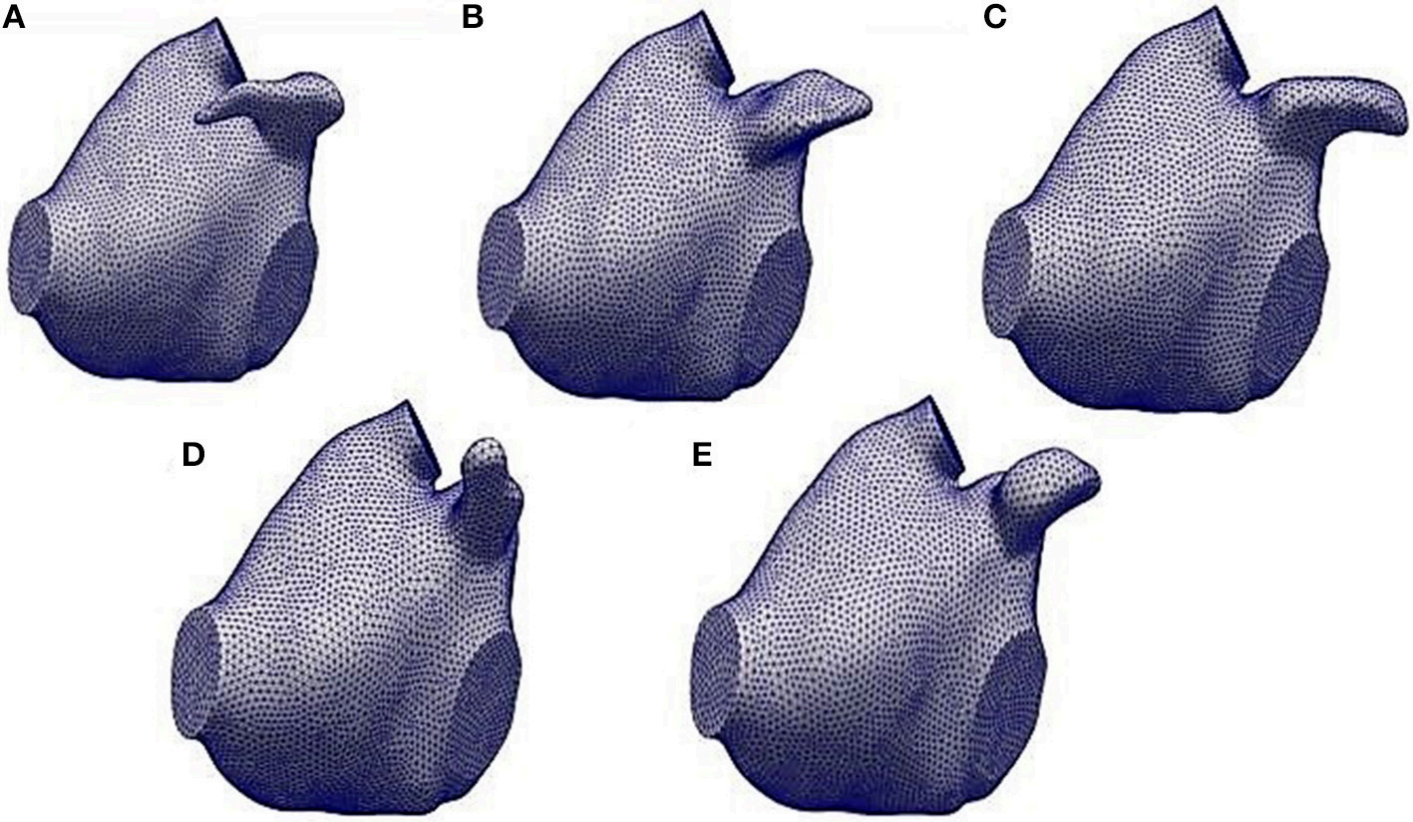
The five LA anatomical models obtained by the automatic framework described in sections 2.2 and 2.3. **(A)** refers to the LA model with the LAA1, **(B)** the one with the LAA2, **(C)** with the LAA3, **(D)** with the LAA4 and **(E)** with the LAA5.

The five anatomical models created by the framework described above are illustrated in Figure 4. The shape and the geometry of the LAAs were different and, in order to quantify the differences between the LAAs and their impact on the haemodynamic characteristics, we performed a specific analysis by quantifying LAA volume, LAA surface area, length, tortuosity, and LAA orifice perimeter and area. Length was evaluated by the LAA centerlines computation. Tortuosity was defined as (Piccinelli et al., 2009):

(1) $$\begin{array}{l} X=\frac{L}{D}-1; \end{array}$$

where L is the centerline length and D is the Euclidean distance between the centerline endpoints. Tortuosity values much higher than 0 reflect the complexity of the LAA shapes, whereas values near to 0 describe more orderly the LAA geometries.

In order to compute the LAA volumes and orifice (i.e., LAA ostium) areas, LAA volume meshes were generated. For this step, the VMTK library was employed (Antiga and Steinman, 2012). The finite element count for each LA mesh is reported in Table 1. The LA volume meshes represented the computational domain of the CFD model.

**Table 1:** Mesh size of each analyzed model.

	**Mesh size (# of finite elements)**
LA model with LAA1	1,878,564
LA model with LAA2	1,767,432
LA model with LAA3	1,923,543
LA model with LAA4	1,698,675
LA model with LAA5	1,716,897

### 2.5. CFD Model

The CFD model used for the simulation of the haemodynamics of each anatomical model was the one presented by Masci et al. (2017) and based on the ℙ1 − ℙ1 finite element method with SUPG-VMS stabilization of the Navier-Stokes equations described in Forti and Dedè (2015). In particular, the blood flow was modeled as a fluid governed by the incompressible Navier-Stokes equations written in the ALE frame of reference (Khurram and Masud, 2006; Reymond et al., 2013; Quarteroni et al., 2017).

Regarding the imposition of the boundary conditions, we considered a mitral valve (MV) flowrate *Q*^0^ from Koizumi et al. (2015). This flowrate was suitably modified for our application: indeed, we removed the atrial contraction wave (A wave) because this is strongly reduced during AF (Gautam and John, 2011). Afterwards, the flowrate at each pulmonary vein (PV) was computed by enforcing mass balance conservation for all *t* ∈ (0, *T*]:

(2) $$\begin{array}{l} Q_{1}^{pv}+Q_{2}^{pv}+Q_{3}^{pv}+Q_{4}^{pv}+Q^{O}+\frac{dV}{dt}=0 \end{array}$$

where $Q_{i}^{pv},\left(i=1,2,3,4\right)$ were the flowrates of each pulmonary veins, *Q*^*O*^ was the flowrate at the MV section and $\frac{dV}{dt}$ is the LA volume variation. From Equation 2, we defined $Q_{tot}^{pv}$, the total flux at the PVs:

(3) $$\begin{array}{l} Q_{tot}^{pv}=Q_{1}^{pv}+Q_{2}^{pv}+Q_{3}^{pv}+Q_{4}^{pv}. \end{array}$$

The total flux $Q_{tot}^{pv}$ through the four pulmonary veins was split with a criterion based on proportionality with their sectional area:

(4) $$\begin{array}{l} Q_{l}^{pv}=\frac{A_{l}}{A_{t}}Q_{tot}^{pv}-Q_{l}^{w}\;l=1,2,3,4, \end{array}$$

where *A*_*l*_ is the sectional area of each PV and *A*_*t*_ is the sum of PVs sectional areas. $Q_{l}^{w}$ represents the term related to each PV section velocity during the cardiac cycle. This term is not null in our application since we applied a random displacement function to our computational domain throughout the cardiac cycle. Therefore, PVs sections are allowed to displace along the heartbeat. In this way, for each *t* ∈ (0, *T*], we were able to evaluate the flowrate at each PV to be applied at the computational model.

In order to limit the presence of unphysical backflows which may give rise to numerical instabilities at the outflow boundary (i.e., MV), we considered the natural-type boundary condition reported in Bazilevs et al. (2009) with backflow penalization.

The numerical discretization of the CFD model was implemented in LifeV, a state-of-art library for the CFD simulations in the parallel setting ^1^. Twenty cores (Intel Xeon processor E5, 2.5 GHz) were used for each LA CFD simulation.

### 2.6. Numerical Simulation and Fluid Dynamics Parameter Computation

For each LA anatomical model, we performed a simulation in AF condition. Unfortunately, due to the requirement of ECG triggering, no imaging technique is available to acquire data during AF episodes from which a realistic and patient-specific deformation model might be derived. Since LA motion in AF is qualitatively defined as an irregular, disorganized, very rapid and strongly reduced contraction, we modeled atrial contraction by employing a random displacement applied independently to each vertex of the anatomical LA model and consisting in a sinusoidal function at a frequency of 4 Hz multiplied by a random factor from an uniform probability density function from 0 to 1. Moreover, the sinusoidal wave was modulated by a small amplitude (0.1 mm) in order to mimic the strongly reduced contraction and avoid numerical issues arising from an excessive worsening of the mesh quality. Simulations were run for five cardiac cycles to avoid the influence of the unphysiological initial condition on fluid velocity. We report the results of the fifth simulated cardiac cycle.

Regarding the parameters of the fluid dynamics model, the time step was set to 0.005 s, dynamic viscosity was 0.035 *poise* and the density was set to 1.06 *g*/*cm*^3^.

The most significant parameters able to describe LAA fluid dynamics including velocity and vortex structures, LAA orifice velocity and mainly the residence time were computed. For the computation of the vortex structures the Q-criterion was employed. It is defined as in the following:

(5) $$\begin{array}{l} Q=\frac{1}{2}\left({\text{W}}_{ij}{\text{W}}_{ij}-{\text{S}}_{ij}{\text{S}}_{ij}\right); \end{array}$$

being ${\text{S}}_{ij}=\left(\frac{\partial {\text{u}}_{f_{i}}}{\partial {\text{x}}_{j}}+\frac{\partial {\text{u}}_{f_{j}}}{\partial {\text{x}}_{i}}\right)$ and ${\text{W}}_{ij}=\left(\frac{\partial {\text{u}}_{f_{i}}}{\partial {\text{x}}_{j}}-\frac{\partial {\text{u}}_{f_{j}}}{\partial {\text{x}}_{i}}\right)$ the symmetric and antisymmetric parts of the velocity-gradient tensor $\frac{\partial {\text{u}}_{f_{i}}}{\partial {\text{x}}_{j}}$. Through this quantity, we identified and visualized some connected regions where *Q* > 0 and the pressure was lower than the ambient value at the vortexes core.

## 3. Results

### 3.1. LAA Shape Descriptors

The values of the computed geometric parameters for each LAA are reported in Table 2.

**Table 2:** LAA geometrical parameters: V, volume; A_*s*_, surface area; A_*o*_, orifice area; P_*o*_, orifice perimeter; L, length; χ, tortuosity.

**LAA**	**V (*cm*^3^**)	**A_*s*_** **(*cm*^2^**)	**A_*o*_** **(*cm*^2^**)	**P**_***o***_ **(*cm*)**	**L (cm)**	**χ**
1	2.04	8.94	1.86	4.98	3.54	0.46
2	2.57	9.25	2.13	5.65	2.26	0.05
3	2.60	10.10	1.87	5.06	2.80	0.08
4	2.21	9.11	1.62	4.90	2.52	0.03
5	2.12	8.31	1.15	3.88	2.36	0.26

Results showed a relevant variability in the LAA characteristics: for example, volume varied between 2.04 and 2.60 cm^3^, length from 2.26 to 3.54 cm and tortuosity values are between 0.03 and 0.46. Comparing these quantities with a qualitative analysis of each LAA from Figure 4, we notice how the geometric indexes reflected the LAA geometric and morphological features. Considering LAA4, we observed, from a purely qualitative point of view, that it presented a very simple geometry and a linear morphology. Indeed, looking at the computation of its geometric indexes, tortuosity was the lowest (0.03) with respect to the other LAAs. As expected, LAA3 showed the highest volume and surface area, as we can see from Figure 4. Moreover, LAA2 had the lowest length despite the major values of LAA orifice perimeter and area. Finally, LAA1 presented the highest tortuosity and length even though its volume was small. Therefore, these results confirmed the high variability of the geometric characteristics of the LAA and it could be relevant quantifying their implications in the appendage fluid dynamics mainly during AF in order to evaluate their impact on the probability of thrombi formation.

### 3.2. Velocity analysis

**Figure 5:**
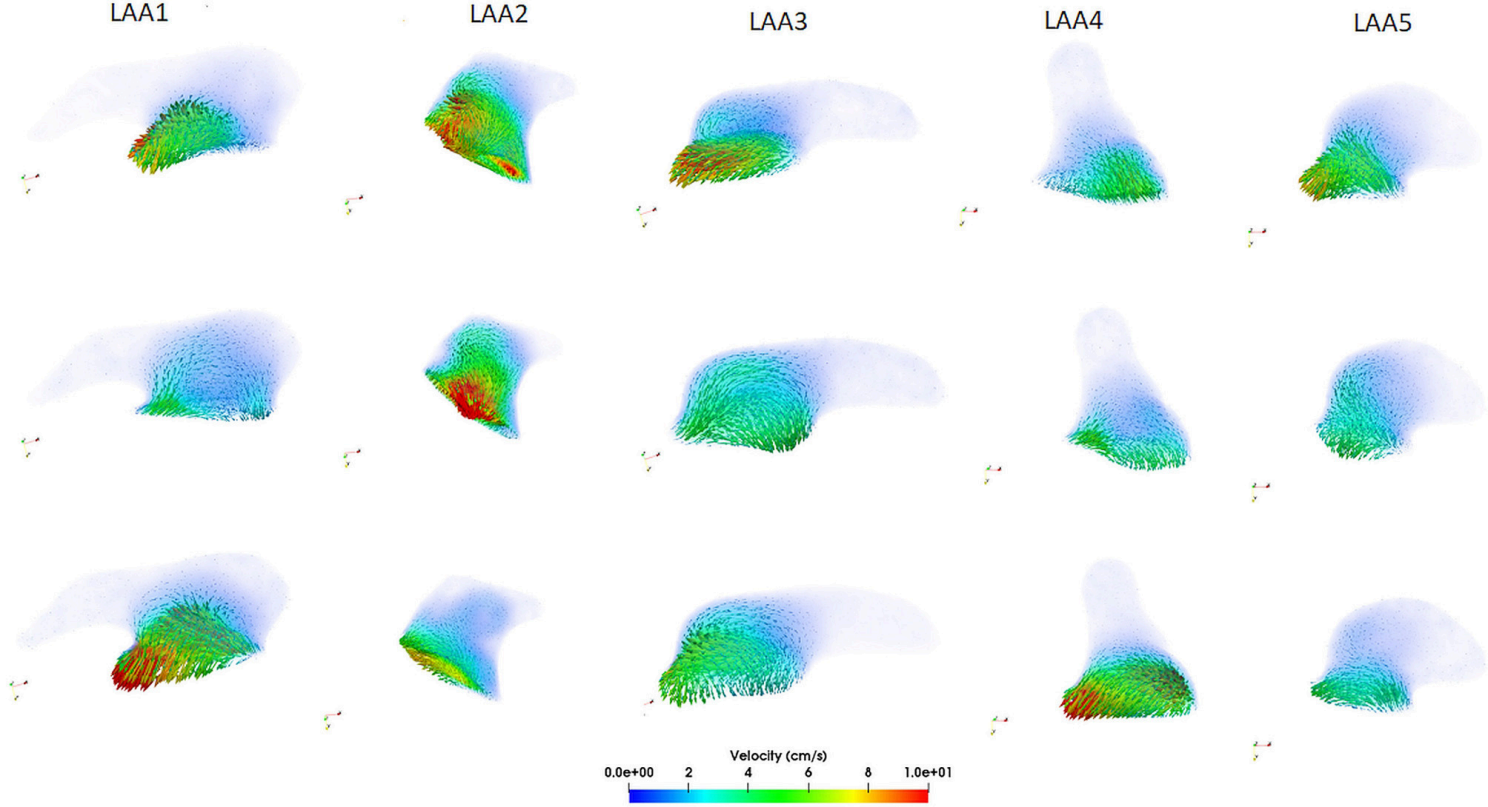
Computed velocity within each LAA during the ventricular systole (first row), at the beginning of the ventricular diastole (second row), and at the end diastole (third row).

The computed velocity from the CFD simulations for the five LAAs is depicted in Figure 5.

Looking at the LAA1, we noticed that the blood flow started to enter into the auricle during the ventricular systole (first row, see Supplemental Video1.mp4) with velocity values between 7 and 9 cm/s. After the end of the ventricular systole the mitral valve opened (second row) and the blood flow exited from the LAA. Velocity was very low in this phase (2–3 cm/s) and these values could be associated with the morphology of the LAA1. Finally, looking at the end diastole phase (third row), blood flow continued to exit from the LAA1, reaching the maximum velocity (10 cm/s). Regarding the LAA2, the shape of this auricle was different with respect to the LAA1 and these differences were reflected by the velocity analysis. These two LAAs had comparable surface areas, orifice perimeters and areas; however volume (V), length (L) and tortuosity (χ) were very different (26 %, 56.6 % and one order of magnitude variations, respectively) and their values helped to elucidate the differences in the hemodynamic parameters. Blood flow entered and came out with a higher velocity than LAA1 (range:15–20 cm/s, see Supplemental Video3.mp4) and a more copious quantity of the blood flow within the LAA2 was noticed. Having a volume higher than LAA1 and a less compexity and length, LAA2 held a great quantity of blood flow which reached also its distal part. Regarding the LAA3, we supposed that, giving its qualitative similarity with the LAA1, also the velocity characteristics should not expect great variability between these two auricles. However, changes in the velocity profile were observed and they could be related to the different geometric parameters with respect to the LAA1. LAA3 presented a higher length than LAA2 but less than LAA1 and χ was one order of magnitude less than LAA1 and double with respect to the LAA2. Therefore, blood flow entered (first row Figure 5, see Supplemental Video5.mp4) and exited (third row Figure 5) from this appendage with intermediate values between LAA1 and LAA2 ( 6-7 cm/s). Moreover, the different orientation with respect to LAA1 allowed the blood flow to get into the auricle more easily. These observations indicated that blood washout for LAAs geometries similar to the LAA3 was not strongly reduced; however it was limited by the complexity of this morphology and also by its non-linear geometry. LAA4 was the most “linear” geometry. Indeed, looking at the Table 2, tortuosity was the smallest with respect to the other auricles. Therefore, we expected that this type of LAA would have a blood flow washout similar to the LAA2. However, even though its simple morphology, we noticed that blood flow, differently from the LAA2, did not reach, throughout the cardiac cycle, the distal parts of this LAA. In order to better understand these findings, we studied the differences in the other geometric parameters. We found that LAA4 was characterized by a particular geometry: it presented a high orifice perimeter and, as we can see from Figure 4, shrinked toward its distal part. Indeed the volume was not very high (2.21 cm^3^). This particular geometry allowed the blood to easily enter in the LAA, giving the large orifice, but it did not penetrate in the more shrinked part of the auricle and it was forced to exit with high velocities ( 10–12 cm/s, see third row of Figure 5, Supplemental Video7.mp4). LAA5 velocity values were much lower than the other appendages and they did not exceed 7 cm/s (see Supplemental Video9.mp4). LAA5 had the smallest values of A_*o*_, V, A_*s*_, P_*o*_ coupled with a high value of the tortuosity (0.26). These characteristics probably explain the difficulty of the blood flow to enter in this type of LAA because of its reduced orifice, despite its length was not very high (2.52 cm). Therefore, only a small quantity of the blood flow entered in LAA5 with low velocity, implying a reduced washout of this auricle comparable to LAA1 and LAA3 but much lower than LAA2.

### 3.3. Vortex Structures Analysis

**Figure 6:**
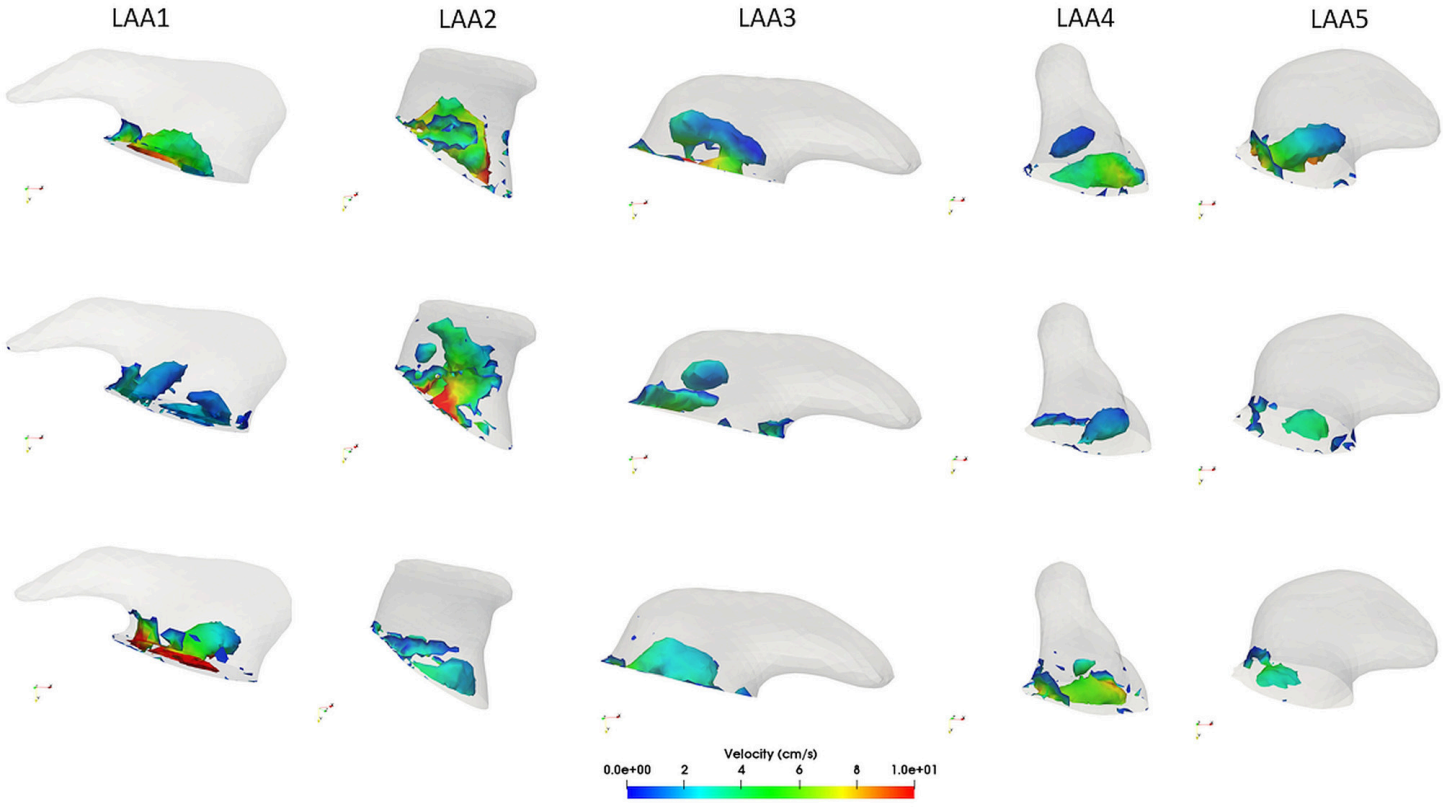
Computed vortex structures with the Q-criterion within each LAA during the ventricular systole (first row), at the beginning of the ventricular diastole (second row) and at the end diastole (third row).

Vortex structures in each LAA are reported in Figure 6. In general, the presence of vortex structures within the LAA may be indicative of a better blood flow washout, thus avoiding the risk of blood stasis. Our considerations focused on the velocity of each LAA were confirmed by the vorticity analysis. LAA1 vortex structures in three different phases of the cardiac cycle are depicted in the first column of Figure 6 (see also Supplemental Video2.mp4). We noticed that most of the vortices were localized in proximity of the LAA orifice, where we found the highest velocity. After the MV opening (second row Figure 6), vortex structures were characterized by low velocities. Moreover, throughout the cardiac cycle, vortices did not reach the distal part of the LAA, thus implying a scarce blood washout in AF. Different findings were observed on the LAA2. Indeed, given its simple morphology, vortex structures with higher velocity values than LAA1 occurred. Moreover, these vortices were not concentrated only in proximity to the LAA ostium and they reached all the anatomical parts of the LAA. (see Supplemental Video4.mp4) Results obtained on the LAA3 (third column, Figure 6, Supplemental Video6.mp4) were similar to the LAA1. Indeed, vortex structures were localized near to the orifice. However, it seemed that these vortices got closer to the LAA tip, favoring a better blood flow washout with respect to the LAA1.

As discussed in the LAA4 velocity profile analysis, we expected that most of the vortex structures are localized in proximity of its orifice. Our hypothesis was confirmed, as shown in the fourth column of Figure 6 (see also Supplemental Video8.mp4). In all the phases of the cardiac cycle vortex structures remained near to the ostium with low velocities. Looking at the LAA5 (see Supplemental Video10.mp4), giving its low velocities throughout the cardiac cycle, also the number of vortex structures was scarce and characterized by small velocities. Moreover, as for the LAA4, they were localized most in proximity of the orifice.

### 3.4. Blood Velocity at the LAA Orifice

**Figure 7:**
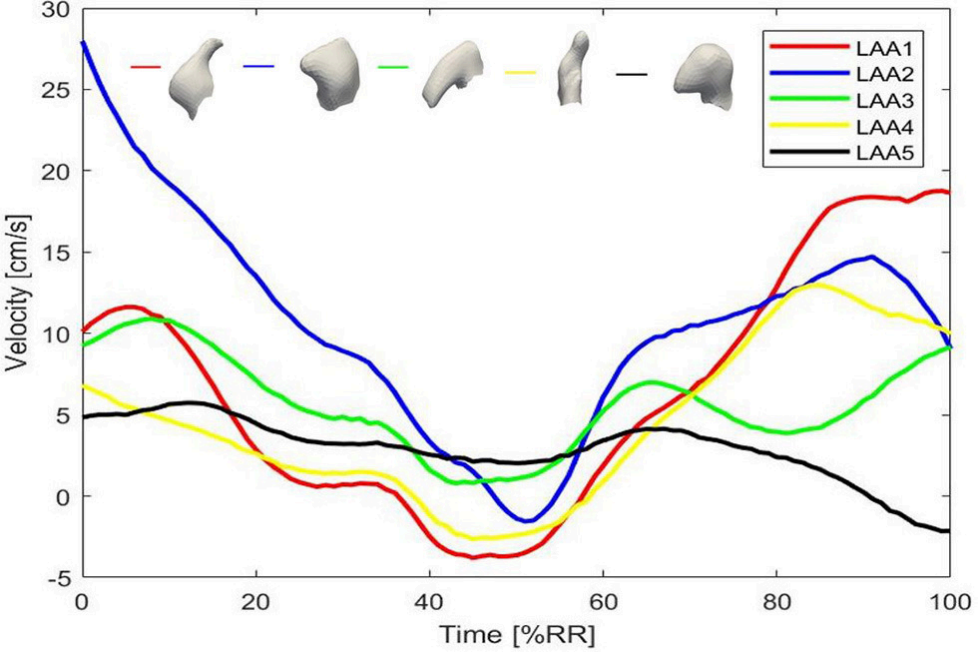
Computed velocity at the LAA ostia for the five LAAs.

The profile of the LAA ostium velocity was evaluated and the results are reported in Figure 7. We considered the last cardiac cycle for each LAA and negative values of the velocity indicated a filling of the LAA, whereas positive values meant LAA emptying. As highlighted in Figure 7, all the auricles were characterized by low values of filling velocity (values between 0 and −5 cm/s). The most relevant result on this analysis was the evaluation of the emptying velocity since it could provide a measure of the washout. We noticed an oscillatory trend in all the appendages and this was probably due to the lack of the atrial contraction imposed in our simulation in order to represent AF condition, which implied a passive LAA emptying and filling. The lack of the contraction of the LA and of the LAA led the orifice velocity not to exceed, except for LAA2, the threshold of 20 cm/s. This data, in agreement with literature (Beigel et al., 2014), implied a confined/circumscribed washout and consequently a possible increase of the thrombogenicity.

Looking at the LAA1, orifice velocity after an initial increase, decreased and reached the lowest value with respect to the other LAAs. This phase was followed by a new increase characterized by a velocity peak of 18.8 cm/s, which was closed to the threshold and could indicate a good washout. However, in the earlier stage the filling occurred with high velocity (absolute value 5 cm/s) and this meant that that part of blood flow could remain in part within the LAA. LAA2 peak velocity was 29 cm/s. Therefore, this evaluation confirmed that within the LAA2 geometry blood flow entered and exited with higher velocity and reached also the tip of the auricle, providing a significant washout. Regarding LAA3 and LAA4, their peak orifice velocity were 10.9 and 13 cm/s, respectively.

LAA5 ostium velocity strengthened our previous findings, indeed it showed a peak value at the ostium of 6 cm/s.

### 3.5. LAA Blood Stasis Quantification

In order to quantify blood stasis in the LAA, we performed a specific study. Each LAA was populated by 500 fluid particles at the beginning of the CFD simulation. The The fluid particles were distributed as a sphere around the center c, the midpoint of the LAA centerline. Paraview software was employed for this analysis (Ahrens et al., 2005). Then, we evaluated how many fluid particles remained within the LAA at the end of the third, fourth and fifth cardiac cycle. The number of residual particles at different timesteps of the cardiac cycle could provide a direct measure of LAA blood stasis: more particles remain in the LAA, higher is the probability of the clot formation within the LAA. Results of this computation are reported in Table 3.

**Table 3:** Number of fluid particles which remained in the LAA at the end of the third, fourth and last cardiac cycle for the five LAAs in AF.

**Cardiac Cycle**	**LAA1**	**LAA2**	**LAA3**	**LAA4**	**LAA5**
0	500	500	500	500	500
3	204	186	266	437	310
4	182	124	133	379	178
5	168	58	103	312	126

*Cardiac cycle = 0 indicates the beginning of the simulation*.

At the end of the fifth cardiac cycle, 33.6 % of fluid particles remained in the LAA1. LAA2 results showed important differences with respect to the LAA1: only the 11.6 % of the fluid particles remained within the auricle. LAA3, as expected, presented a better washout with respect to the LAA1. Indeed, 20.6 % of fluid particles did not exit from the LAA. Despite their similarity in shape, the difference between LAA1 and LAA3 could be caused by the opposite orientation and the different value of tortuosity.

62.4 % of fluid particles remained within the LAA4 because the blood flow pushed the particles to the tip and velocities in this part were very low.

The blood washout of the LAA5 was not strongly reduced: 25.2 % of particles remained within the appendage.

## 4. Discussion and Conclusion

In this study, we assessed the influence of the LAA morphology on the LA hemodynamics. The framework developed and described in this study is fully automatic and fast. In our experience this procedure is very robust since it worked correctly for all our data; it requires < 1 min to generate one models and most of this time is required to import the mesh and compute the shape diameter function. Several geomeric parameters were computed and correlated with blood velocities, vorticity, LAA ostium velocity and residence time for each anatomical model. We found the LAA geometric characteristics impact on the hemodyamic pattern within the LAA highlighting that not only the appendage morphology types should be considered for the stroke risk assessment in patients affected by AF (Di Biase et al., 2012). Our results on the velocity and vorticity within the LAA, LAA orifice velocity and on the residence time demonstrated and confirmed that not only complex LAA morphologies were characterized by low velocities, low vorticity and consequently a higher thrombogenic risk. Simple morphologies can have a thrombogenic risk equal, or even higher, than more complex auricles and their geometric features could play a key role in defining thromboembolic risk. Indeed, in our opinion, LAA geometric parameters should be considered, coupled with the morphological characteristics, for a comprehensive evaluation of the blood stasis and stroke risk. These geometric characteristics were not investigated in the previous works, neither correlated with hemodynamic parameters.

LAA1 presented the highest length (3.54 cm) and tortuosity (0.46), thus representing a complex shape. Indeed, the quantity of the blood flow which reached the distal part of the LAA1 throughout the cardiac cycle was strongly reduced due to the complex morphology of this LAA, as explained in section 3.2. Moreover, vortex structures within this appendage were limited in number and did not reach its distal part. Since Beigel et al. (2014), observed that the stroke risk is 2.6 fold higher in AF patients in which the velocity at the ostium throughout the cardiac cycle is <20 cm/s and these findings were confirmed in the CFD studies on the LAA in AF by Zhang and Gay (2008); Olivares et al. (2017), we also evaluated the LAA orifice velocity. LAA1 ostium velocity was lower than 20 cm/s, indicating a high trombogenicity. LAA1 blood stasis quantification confirmed this result, showing 33.6 % of fluid particles remained within the LAA1. Fluid particles were pushed toward the appendage tip and the low blood flow velocity hampered an effective washout. Therefore, we expect that LAA geometries similar to LAA1 may have an high thromembolic risk.

Study of LAA2 fluid dynamics indicators proved that linear structures, coupled with a small length, had a better washout, thus implying a reduction of the blood stasis and thrombi formation risk. Moreover, the positioning of the LAA2 could have a key role for the stroke risk assessment. As highlighted in Figure 4, LAA1 had an opposite positioning with respect to the others. This characteristic could explain the scarce washout, mainly in the distal part, thus increasing the probability of thrombi formation risk. The LAA2 blood stasis analysis confirmed this consideration: indeed the simple geometry allowed and helped the blood flow to reach the tip of the LAA and therefore to fill the appendage volume. We concluded that the probability of clot formation for the auricles which present a morphology similar to the LAA2 is low.

LAA3, despite its qualitative similarity with the LAA1, presented relevant differences in the hemodynamic parameters. Velocities were slightly higher than LAA1 and blood flow entered within this auricle more easily and consequently vortex structures were deeper. Moreover, looking at the number of fluid particles which remained in the LAA3 at the end of the fifth cardiac cycle, we found that LAA3 had a better washout than LAA1. Hemodynamic differences between these two auricles could be related to the different orientation and tortuosity, as explained in section 3.2. Therefore, the thrombogenicity risk of this type of LAA could be classified as medium.

LAA4 velocity profile highlighted that for the assessment of left atrial appendage thrombogenicity, geometric and morphological features should be take into account. Indeed, as reported in the velocity field analysis, even qualitatively simple LAAs (i.e., LAA4) could show a high probability of blood stasis and therefore thrombi formation. LAA4 vortex structures were localized in proximity of its orifice implying that blood washout in the distal parts of this auricle was scarce, thus increasing the probability of clot formation with respect to the other LAAs. Yet, we found low velocity values at the ostium in the LAA3 and LAA4, thus indicating a moderate clot formation risk for this particular LAA shape. In order to confirm our previous findings, we quantified the LAA4 blood stasis. The fluid did not reach the auricle tip because of the shrinking toward the distal part. These characteristics led to a scarce washout of the LAA4 (see Table 3), moreover the vortex structures were absent, thus implying a blood stasis in proximity of the LAA4 tip. This result was very relevant because it proved that the LAA4 showed the highest probability of blood stasis and consequently of thrombi formation with respect to the other appendages, despite its simple and linear geometry, where a low thrombogenicity was expected. Regarding the LAA5, velocity within the auricle and at the orifice showed low values that could imply a relevant risk of blood stasis and thrombi formation. Also, vortex structures were localized only in proximity of the LAA orifice. However, even though the velocities and the number of vortex structures were very small, the blood washout of the LAA5 was not strongly reduced. LAA5 limited extension could compensate the high tortuosity, allowing the blood flow to entirely cover its volume, thus providing a good washout.

The few CFD studies available on this topic (Koizumi et al., 2015; Otani et al., 2016; Olivares et al., 2017; Bosi et al., 2018) focused on the correlation between the four type of the LAA morphologies proposed by Di Biase et al. (2012) and the thrombogenicity. However, they did not analyze other characteristics of the auricles that might have a crucial role in pathological conditions. With respect to the other works, we designed and developed a procedure which allowed to consider the same LA shape for each LAA. To perform this task, the SDF was employed. Most of previous segmentation approaches were based on local surface properties, such as curvature or geodesic distance, that often depend on the topology and on the pose of the object. SDF overcame these problems and provided a link between the surface mesh and the object's volume, thus allowing the extraction a specific part of the mesh. We chose this option because the variability of the left atrial chamber could affect the validity of the hemodynamics changes when different types of LAA were compared, since these variations could be caused also by the LA geometric features (dimensions, structure, and pulmonary vein attachments and morphology). Obviously the clinical problem we are facing is very complex and we think there may be an interchangeable conditioning effect between the LA and the LAA shapes and both effects should be considered. However, we also think the comprehension of each single effect may help in clarifying the interplay between them. In this study we focused on better understanding the influence of each specific LAA shape on blood hemodynamics and, to this aim, we were forced to eliminate the dependence from the LA shape and from other patient-specific factors. Our results, based on a simplification of the real phenomenon, showed that the complexity of the LAA shape alone does not correlate with clot formation and additional parameters should be considered for a clear comprehension of the link between LAA shape and the risk of stroke.

Our approach can be further improved because a limitation consists in the number of the available left atrial data-set. Future developments will be focused on considering a larger number of patient-specific LAAs in order to fully its wide anatomical variability. Results on a larger dataset would also allow to link the modeling results to thrombogenic risk potentially identifying clinically measurable parameters that may inform as to which treatment option would be best for a particular patient. Unfortunately, for the patients analyzed in this study this information was not available. In addition, the results of this study could benefit from the application of a patient-specific motion field of the LA in AF. Unfortunately, up to date, quantification of such a motion field is not possible using the standard 3D acquisition, MRI or CT. Since AF is described by a disorganized and reduced motion of the LA, in this study we applied a random displacement field with small amplitudes in order to avoid mesh degeneration during simulations. Once the patient-specific motion in AF has become available, our pipeline would strongly benefit from such information. Another possibility is to build a FSI model in which the control of the motion of the computational domain throughout the cardiac cycle could be more realistic than using a random displacement function as the one employed in this study.

To conclude, the presented framework might represent a step toward the development of a better tool for the patient-specific cardioembolic risk assessment and preventive treatment in AF patients.

## Author Contributions

AM: Conception of the study, software development, simulations, analysis of the results, manuscript drafting, figure, and video preparation, revision of manuscript. LB: Software development, simulations. LD: Analysis of the results, revision of manuscript. CT: Image acquisition. MF: Simulator set up. AQ: Simulator design, analysis of the results, revision of manuscript. CC: Conception of the study, analysis of the results, manuscript drafting, revision of manuscript, supervision of the study.

### Conflict of Interest Statement

The authors declare that the research was conducted in the absence of any commercial or financial relationships that could be construed as a potential conflict of interest.

## Footnotes

^1^www.lifev.org
