# Supplementary material for: The Impact of Left Atrium Appendage Morphology on Stroke Risk Assessment in Atrial Fibrillation: A Computational Fluid Dynamics Study
Source: Front Physiol. 2019 Jan 22;9:1938. doi: 10.3389/fphys.2018.01938 (PMC6349592; doi:10.3389/fphys.2018.01938)
Supplement: Supplementary file 11 [file Data_Sheet_1.PDF]

frontiers

Supplementary Material

The impact of LAA morphology on stroke risk assessment in atrial fibrillation: a computational fluid dynamics study

Alessandro Masci[1]*, Lorenzo Barone[1], Luca Dedè[2], Marco Fedele[2], Corrado Tomasi[3], Alfio Quarteroni[2], Cristiana Corsi[1]

[1] DEI, University of Bologna, Campus of Cesena, Bologna, Italy

[2] MOX, Mathematics Department, Politecnico di Milano, Milano, Italy

[3] Department of Cardiology, Santa Maria delle Croci Hospital, AUSL della Romagna, Ravenna, Italy

*** Correspondence:** Alessandro Masci: alessandro.masci4@unibo.it; Cristiana Corsi: cristiana.corsi3@unibo.it

Supplementary Data

Supplemental video: LAA1_vel.mp4

Supplemental video: LAA1_vort.mp4

Supplemental video: LAA2_vel.mp4

Supplemental video: LAA2_vort.mp4

Supplemental video: LAA3_vel.mp4

Supplemental video: LAA3_vort.mp4

Supplemental video: LAA4_vel.mp4

Supplemental video: LAA4_vort.mp4

Supplemental video: LAA5_vel.mp4

Supplemental video: LAA5_vort.mp4
